# Baby Week

**Published:** 1920-05-01

**Authors:** 


### Baby Week.

BABY WEEK, organised by the National Baby Week Council (of which the Queen is patron, the Prime Minister President, and Viscount Astor Chairman) will be from July 1 to July 7, and its general object is the necessary one of arousing the public to a sense of the importance of infant life, and, broadly, of extending the influence of all the agencies bearing upon it. It is suggested that local committees should be formed, including representatives of the local authorities, the medical profession, nurses, welfare workers, etc., and that they should arrange for the help of the Press, of the churches, and of the trade unions; organise public meetings, lantern-lectures, mothercraft exhibitions, local competitions, dramatic performances dealing with mother and child life, baby shows (with special care), and other means of spreading information.

One thing which should be urged during this Baby Week is the absolute urgency of the milk problem. The price is certainly a little lower, but there is much to be desired regarding purity. The Government, we are told, will shortly introduce a comprehensive pure-milk Bill, and all efforts should be made to secure that it is in reality a pure-milk Bill. We are still a long way off pure milk, and whatever legislation there is, let it at least be effectual. The Profiteering Act is so sorry an example of futility that we may be excused the absence of lively enthusiasm in advance for the Government measure foreshadowed.

